# Book review – Global Bioethics: an Introduction by Henk ten Have

**DOI:** 10.3325/cmj.2016.57.402

**Published:** 2016-08

**Authors:** Ana Borovečki

**Affiliations:** School of Medicine, University of Zagreb, Zagreb, Croatia

When one googles the words “global bioethics” and “the book,” one can see that the author of the greatest number of books with “global bioethics” in the title is Henk ten Have. After a handbook and an encyclopedia of global bioethics and after a discussion on bioethics education from a global perspective, Henk ten Have decided to write an introduction to the subject of global bioethics. His latest book, Global Bioethics: an Introduction (1) gives an interesting historical overview of the bioethics development, clearly describing all the phases in its development, from the Hippocratic tradition and traditional medical ethics to bioethics and global bioethics. For him, bioethics as we know it today, with issues that emerge in everyday medical practice, like beginning and the end of life, allocation of resources, physician-patient relationship, is in the new phase of its evolution – the phase of global bioethical issues and global bioethics. He states “The global dimension of bioethical issues had produced a new stage, or a new kind of bioethics. …..Global bioethics implies the quest for an encompassing ethical framework that applies globally.” The aim of this book is to explain what global bioethics is. He is aware of all the pitfalls in his journey of describing the field, of all versions of global bioethics: “thin versions,” “thick versions,” and “intermediate versions” as he calls them. “Thin versions” authors agree that the term “global bioethics” can be used to describe new problems emerging in the field of bioethics, global problems or problems with a global dimension, but they do not think that this is a special approach to bioethics. “Thick versions” scholars are aware that global bioethics is substantive and comprehensive bioethics that articulates ethical perspectives relevant worldwide. However, the issue of overcoming moral pluralism in the world inhabited by moral strangers, where ethical values are related to specific communities, lingers over the prospect of “thick versions” approach. Global bioethics in “intermediate versions” is at the same time connected to a universal ethical framework based on intercultural dialogue and consensus and still applicable in diverse cultural settings. “If there are bioethical values and principles that are universal in scope their meaning has to be specified in particular contexts before they can be applied to the issues and problems arising there.” states ten Have. It is clear that ten Have is opting for the intermediate version of bioethics and he elaborates a practical implementation of his approach to global bioethics. He clearly describes global bioethical problems and provides us with frameworks and common perspectives, and finally talks about global bioethical discourse, not omitting the importance of global health governance and bioethics governance. Henk ten Have, in 272 pages of his book, gives a well described framework of global bioethics, in a clear, understandable, and interesting manner, using a plethora of up-to-date examples in order to demonstrate his approach to global bioethics. He made things even clearer with a summary at the end of each chapter and a useful index at the end of the book. If you want to understand what global bioethics is, how it all began, and what its future brings, this is a book for you to read.
